# Tree diversity drives associational resistance to herbivory at both forest edge and interior

**DOI:** 10.1002/ece3.5450

**Published:** 2019-07-18

**Authors:** Virginie Guyot, Hervé Jactel, Baptiste Imbaud, Laurent Burnel, Bastien Castagneyrol, Wilfried Heinz, Marc Deconchat, Aude Vialatte

**Affiliations:** ^1^ DYNAFOR, INRA, Université de Toulouse Castanet Tolosan France; ^2^ BIOGECO, INRA, Univ. Bordeaux Cestas France; ^3^ LTSER Zone Atelier «PYRÉNÉES GARONNE» Auzeville‐Tolosane France

**Keywords:** ecosystem functioning, forest edge, insect herbivory, plant diversity

## Abstract

Tree diversity is increasingly acknowledged as an important driver of insect herbivory. However, there is still a debate about the direction of associational effects that can range from associational resistance (i.e., less damage in mixed stands than in monocultures) to the opposite, associational susceptibility. Discrepancies among published studies may be due to the overlooked effect of spatially dependent processes such as tree location within forests. We addressed this issue by measuring crown defoliation and leaf damage made by different guilds of insect herbivores on oaks growing among conspecific versus heterospecific neighbors at forest edges versus interior, in two closed sites in SW France forests. Overall, oaks were significantly less defoliated among heterospecific neighbors (i.e., associational resistance), at both forest edge and interior. At the leaf level, guild diversity and leaf miner herbivory significantly increased with tree diversity regardless of oak location within stands. Other guilds showed no clear response to tree diversity or oak location. We showed that herbivore response to tree diversity varied among insect feeding guilds but not between forest edges and interior, with inconsistent patterns between sites. Importantly, we show that oaks were more defoliated in pure oak plots than in mixed plots at both edge and forest interior and that, on average, defoliation decreased with increasing tree diversity from one to seven species. We conclude that edge conditions could be interacting with tree diversity to regulate insect defoliation, but future investigations are needed to integrate them into the management of temperate forests, notably by better understanding the role of the landscape context.

## 1 INTRODUCTION

Within the general biodiversity—ecosystem functioning framework, a large body of research has been addressing associational effects of plant diversity on resistance to insect herbivores (Jactel et al., 2017; Moreira, Abdala‐Roberts, Rasmann, Castagneyrol, & Mooney, 2016). Meta‐analyses showed an overall lower level of insect damage in more diverse plant communities, both in agricultural (Letourneau et al., 2011) and forest ecosystems (Castagneyrol, Jactel, Vacher, Brockerhoff, & Koricheva, 2014; Jactel & Brockerhoff, 2007). Still, this general pattern masks a large variation in the magnitude but also in the direction of associational effects identified in the literature, particularly in forest ecosystems, from positive (i.e., associational resistance, *AR*; Barbosa et al., 2009), neutral (e.g., Haase et al., 2015) to negative effects (i.e., associational susceptibility, *AS*; Schuldt et al., 2010). Current knowledge about mechanisms driving associational effects in plants is largely derived from controlled experiments and has been more commonly addressed in grasslands than in forests. (Grossman et al., 2018; Meyer et al., 2017). Although such experiments perfectly control for plant richness and composition, they are designed to minimize other sources of variation in plant‐herbivore interactions like spatial variability. Yet, a better understanding of ecological drivers of these interactions in real‐world ecosystems requires taking such spatial effects into account.

At a time when the length of forest edges is sharply increasing due to fragmentation associated with road constructions, agricultural intensification, forest logging and housing development (Fahrig, 2003), the risk of forest pest damage is also increasing due to higher recruitment of colonizing herbivores (Didham, Ghazoul, Stork, & Davis, 1996), warmer temperature (due to sunlight) favoring poikilothermic organisms (Kouki, McCullough, & Marshall, 1997; Saunders, Hobbs, & Margules, 1991), or higher probability of abiotic disturbance like wind throw benefiting wood damaging insects (Peltonen, 1999). Forest fragmentation has well documented effects on insect herbivores through increased length of edges and sharp contrasts between edges and interiors of forest fragments (Batary, Fronczek, Normann, Scherber, & Tscharntke, 2014; Fahrig, 2003; Harper et al., 2005; Vodka & Cizek, 2013; Wirth, Meyer, Leal, & Tabarelli, 2008). In particular, the species richness and composition of insect communities differ between forest edges versus interior (Barbosa, Leal, Iannuzzi, & Almeida‐Cortez, 2005; Normann, Tscharntke, & Scherber, 2016; Pryke & Samways, 2011; Souza, Santos, Oliveira, & Tabarelli, 2016). In addition, insect herbivory is generally greater at forest edges as compared to forest interior (De Carvalho, Rodrigues Viana, & Cornelissen, 2014; Maguire, Buddle, & Bennett, 2016; Thompson, Grayson, & Johnson, 2016). Some authors have proposed that this pattern is partially driven by increased abundance and diversity of plant resources and greater proportion of generalist herbivores at forest edges (De Carvalho et al., 2014; Rossetti, Tscharntke, Aguilar, & Batary, 2017). Yet, tree diversity generally triggers associational resistance against specialist herbivore species while effects on generalist herbivore species are generally more variable (Castagneyrol, Jactel, Vacher, et al., 2014). It is therefore likely that the strength and direction of associational effects vary between forest edges and forest interior, which may have profound implication for the dynamic of forest fragments. Yet, to the best of our knowledge, this possibility has rarely been addressed so far (but see van Schrojenstein Lantman et al., 2018).

Tree species diversity has also different effects on different insect feeding guilds (Castagneyrol, Giffard, Péré, & Jactel, 2013; Vehviläinen, Koricheva, & Ruohomäki, 2007). Indeed, associational resistance depends on several biotic and abiotic factors such as host specificity, local climate or bottom‐up and top‐down processes which appear acting differently on different herbivores (Barton et al., 2015; Singer et al., 2014). Importantly, these processes may also be affected by edge effects. First, different herbivore species may respond differently to forest edges (Ewers & Didham, 2006; Ries, Fletcher, Battin, & Sisk, 2004) depending on their traits, *for example,* those driving dispersal and foraging behaviors. Second, differences in abiotic factors between forest edges and forest interior drive changes in leaf traits (Silva & Simonetti, 2009), which may have cascading effects on herbivores (Bagchi, Brown, Elphick, Wagner, & Singer, 2018). Third, the activity of predators also differs between forest edges and interior (Bagchi et al., 2018; Maguire, Nicole, Buddle, & Bennett, 2015; Pryke & Samways, 2011; Ries et al., 2004), thus leading to a differential top‐down control of insect herbivores between forest edges and forest interiors. Altogether, these findings suggest that tree location within forests (i.e., edge vs. interior) may affect associational effects in a way that differs among insect herbivores.

**Figure 1:**
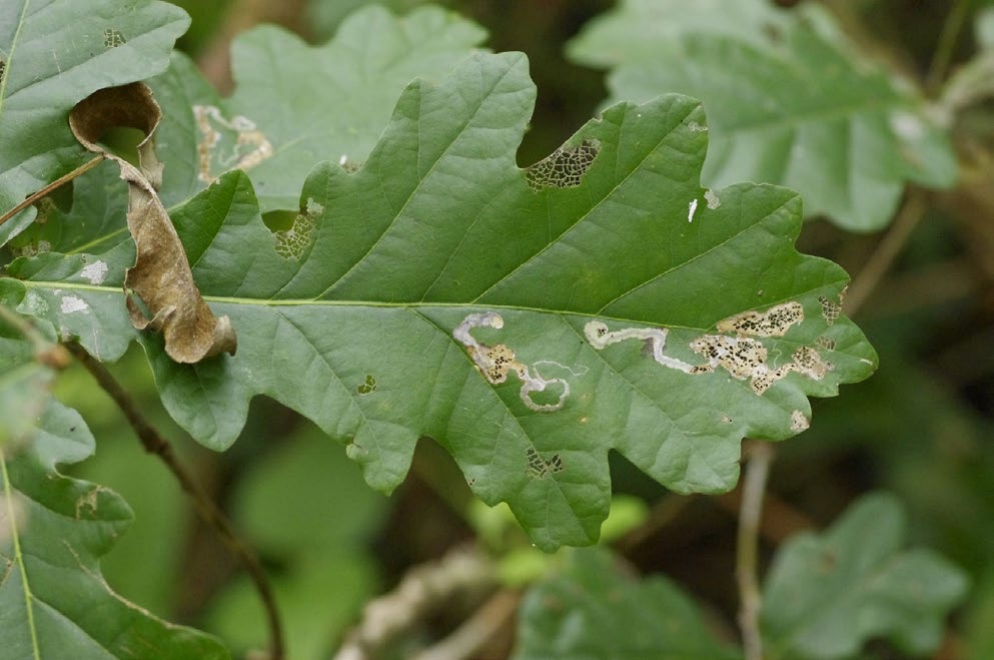
Example of oak leaf presenting damage made by different insect feeding guilds (incl. leaf chewers, skeletonizers and miners)

The main objective of our study was to compare the effect of tree species diversity on insect damage at forest edge versus interior for the whole community of herbivores (measured as total crown defoliation, *for example,* Guyot, Castagneyrol, Vialatte, Deconchat, & Jactel, 2016) and for specific feeding guilds of insect herbivores (Figure 1). We focused on oaks as target tree species and used a complete factorial design, sampling individual oak trees with conspecific versus heterospecific neighbors (hereafter referred to as pure and mixed plots) at both edge and interior of the same forest patches.

## 2 MATERIALS AND METHODS

### 2.1 Study sites

**Figure 2:**
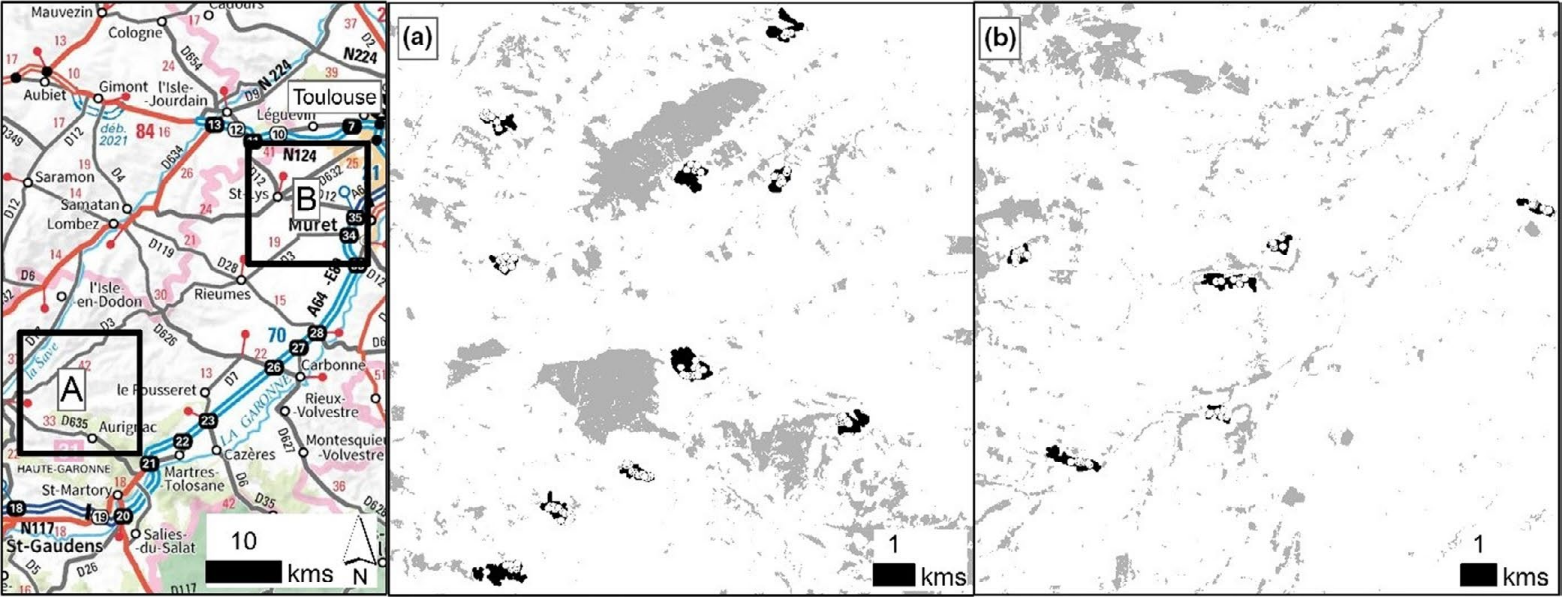
Study sites near Toulouse (SW France). Forest cover is presented for each site in gray (A for Aurignac and B for Lamothe). Forest patches that were studied are in black; white dots in these forest patches represent sampled plots

The study was carried out in forest patches located in the valleys and hillsides of Gascony, a rural landscape of South‐Western France. The climate is temperate with oceanic and Mediterranean influences and soils are mainly calcareous or molasses. Forest patches are dominated by oaks (*Quercus petraea* Liebl., *Quercus robur* L. and *Quercus pubescens* Willd.) mixed with other native deciduous species (*Carpinus betulus* L., *Prunus avium* (L.) L., *Acer campestre* L., *Fraxinus excelsior* L. and *Sorbus torminalis* L. (Crantz)). Sampled plots were located in two close sites, Aurignac and Lamothe (260 km^2^ each) 40 km apart from one another, where forest cover was 18% and 9% respectively (Figure 2, Table 1).

**Table 1:** Characteristics of study sites with number of sampled forest patches, plots and neighboring trees

Study sites	Aurignac	Saint‐Lys	Total
GPS coordinates	43°16′11.6″N	43°30′40.0″N	
	0°50′50.3″E	1°11′30.0″E	
Site elevation (mean)	323 m (±44)	201 m (±28)	
Forest cover	18.5%	9.2%	
Number of sampled forest patches	10	6	16
Number of sampled plots			
Pure			
Edge	13	2	15
Interior	11	6	17
Mixed			
Edge	22	13	35
Interior	24	15	39
Total	70	36	106
Number of neighboring trees	730	376	1,106

### 2.2 Plot selection in forest patches

We established a total of 106 plots, within 16 forest patches (Table 1), between April and October 2012, with the agreements of forest owners. Patch surface area varied between 16 and 46 ha. Within each patch, we aimed at establishing at least four plots: one pure and one mixed plots, both at the edge of and within the patch. A sampling plot (appr. 200 m^2^) consisted of a focal oak tree surrounded by its closest neighboring trees, i.e., with no more than 3 m between neighboring tree crown and focal tree crown. According to the patch area and the distribution of oak species, most patches had more than four experimental plots, while a few had less, resulting in an unbalanced number of replicates per modality of plot diversity × location (Table 1). Neighboring trees were either of the same oak species, i.e., pure plot, or of different trees species, i.e., mixed plots (in order of frequency: *C. betulus*, *P. avium*, *Q. pubescens*, *A. campestre*, *F. excelsior*, *Populus tremula* L., *Robinia pseudoacacia* L., *Castanea sativa* Mill., *S. torminalis*, *Fagus sylvatica* L., *Ulmus minor* Mill., *Pseudotsuga menziesii* (Mirb.) Franco, *Tilia platyphyllos* Scop., *Quercus rubra* L., *Corylus avellana* L., *Crataegus monogyna* Jacq., *Sorbus domestica* L., *Alnus glutinosa* (L.) Gaertn., *Betula pendula* Roth, *Fraxinus angustifolia* Vahl, *Malus sylvestris* Mill. and *Pinus pinaster* Aiton). Tree species richness ranged from 3 to 7 species in mixed plots. Edge plots were located within a 30 m distance from the border of the patch. This threshold distance was used to make sure that focal trees were under an edge influence (Harper et al., 2005; Alignier & Deconchat, 2011). However, the focal tree of edge plots was not right at the edge of the patch, so as to be surrounded by other trees. Interior plots were located in the inner area of the patch, at least 60 m far from the border. The adjacent land cover of forest patches was temporary grassland or annual crop field. The selected forest patches had no large forest roads, clearings or recent cuttings in order to avoid internal edge effects. The sampling design therefore resulted in two orthogonal factors (*Location* and *Diversity*), with two levels each.

To be included in the survey, focal oak trees had to fulfill four criteria, i.e., to be (a) *Q. petraea* or *Q. robur* (we did not distinguish between the two oak species because they are closely‐related species that can hybridize and are therefore difficult to distinguish in the field; furthermore they were assumed to be functionally equivalent in terms of traits involved in oak‐herbivore relationship [Southwood, Wint, Kennedy, & Greenwood, 2004]), (b) dominant or codominant in the canopy (i.e., tree height compared to other trees of the stand) in order to standardize for the tree size, (c) surrounded at 360° by other trees in order to get standardized (symmetrical) crown shapes, and (d) at least 50 m from another sampled focal tree for the sake of independency. A tree was considered a neighbor of a focal oak if (a) its crown was at a maximum of 3 m away from the crown of the focal tree; (b) its diameter at breast height (DBH) was larger than 10 cm; and (c) its height was greater than half the average height of the canopy (in order to exclude too small individuals, including saplings).

The total sample of trees consisted in 106 focal oak trees (i.e., 106 experimental plots) and 1,106 neighboring trees (Table 1), i.e., each focal tree was surrounded by ca. 10 neighboring trees.

### 2.3 Crown defoliation assessment

Crown defoliation, i.e., foliar loss, in focal trees was estimated by adapting the ICP Forests protocol (Eichhorn et al., 2010). One of the main differences was that insect damage was assessed on the whole crown, instead of the “assessable crown” only (see Guyot et al., 2015). To assess crown defoliation, a comparison was made between the focal tree and a reference tree, i.e., a healthy tree with full foliage in the same forest patch. In our protocol, tree crown was separated in two sections, one exposed to sunlight and the other in the shade, as foliar loss may be also due to competition for light or natural pruning in the shaded part, given that oak trees are heliophilous. The assessment was done with binoculars by the same trained person (LB) in order to avoid observer bias.

On each focal oak, the observer visually estimated the proportion of (a) crown volume exposed to sunlight (*P*_CL_), (b) dead branches in the two sections of the crown (*P*_DBL_ for light exposed and *P*_DBS_ for the shady section, respectively) and (c) defoliation in the two sections of the living crown i.e., the crown excluding dead branches (*P*_DefL_ for the sun light exposed and *P*_DefS_ for the shady section, respectively). To estimate the proportion of dead branches in each part of the crown, the total number of branches was counted. The following percentage classes were used for all proportion variables: 0%, >0%–1%, >1%–12.5%, >12.5%–25%, >25%–50%, >50%–75% and >75%. The crown was systematically assessed from two opposite points of view to account for total crown defoliation. The mean of damage class medians (i.e., medians of the two estimates for the two sides per tree) was used if a different score was attributed for different sides of the crown. The total percentage of crown defoliation *T*_Def _was then estimated as:

(1) $$T_{\text{Def}}=P_{\text{ACL}}\times P_{\text{DefL}}+\left(1-P_{\text{ACL}}\right)\times P_{\text{DefS}}$$

where *P*_ACL_ represents the proportion of the living crown exposed to sunlight:

(2) $$P_{\text{ACL}}=\frac{P_{\text{CL}}\left(1-P_{\text{DBL}}\right)}{P_{\text{CL}}\left(1-P_{\text{DBL}}\right)+\left(1-P_{\text{CL}}\right)\left(1-P_{\text{DBS}}\right)}$$

### 2.4 Leaf damage assessment

All focal oak trees were climbed to collect leaf samples from September 9th to 26th, 2013 (with the agreement of forest owners). Two branches were cut at random, one at the top and another one in the middle of tree crown, to obtain a leaf sample on each section of the crown (i.e., sun exposed and shady). On each branch, 50 leaves were collected at random and frozen at −18°C until damage assessment. Damage by seven different feeding guilds was visually assessed by a single person (BI). For leaf chewers and skeletonizers, we scored damage using seven classes of damage (0%, >0%–5%, >5%–10%, >10%–25%, >25%–50%, >50–75, >75%). Chewing damage was assessed first, then skeletonizing damage was assessed on the remaining intact leaf area (Johnson, Bertrand, & Turcotte, 2016). For miners, rollers, tiers, gall makers and sap feeders, we counted the number of leaves with at least one individual damage. The mean percentage of leaf area removed (*defoliation*) by chewers and skeletonizers and the percentage of leaves impacted by each of the other guilds (*incidence*) were calculated for each sampled tree.

### 2.5 Statistical analyses

To test the representativeness of crown assessment we first calculated Pearson's correlations between *T*_Def_ and each insect guild damage estimated with the leaf sample collected in the same focal oak trees.

For each response variable (total crown defoliation *T*_Def_, guild diversity using a Shannon index and guild‐specific damage or abundance), we first built a beyond optimal linear mixed effect model including Site (Aurignac vs. Lamothe), Tree diversity (Pure vs. Mixed stands), Location (forest interior vs. forest edge) as fixed effects as well as every two‐ways interactions. We declared the forest patch (*n* = 16) as a random factor to account for variance arising from non‐independent plots within the same patch. Data on leaf miners, leaf gallers, leaf tiers, leaf rollers and sap feeders were recorded as count data. For these response variables, we used generalized mixed effect models with a Poisson error family and log‐link. In a second model, we replaced the categorical factor plot diversity (pure vs. mixed) by actual tree species richness as continuous variable (ranging from 1 to 7 tree species). Models were built using *lmer* function in *lme4* package (Bates, Mächler, Bolker, & Walker, 2015) in *R* version 3.4.4 (2018‐03‐15).

For each response variable, we applied model selection based on information theory. We ranked the 18 resulting models according to their Akaike's Information Criterion corrected for small sample size (AICc) and calculated the difference between model AICc and the AICc of the best model, i.e., the model with the lowest AICc. According to our sample size, models with ΔAICc < 2 can be interpreted as competing models with no evidence for one being better than the other(s) (Burnham & Anderson, 2002). We also calculated model *R* ^2^ to estimate model fit, and AICc weight. We calculated variable importance as the sum of AICc weights of every models containing this variable as a predictor. Variable importance corresponds to the probability that a given variable is included in the best model (Burnham & Anderson, 2002; Symonds & Moussalli, 2011). However, it does not represent the probability that an explanatory variable is a good predictor of the response variable. We therefore estimated model parameter coefficients and their 95% CI using model averaging. Model comparison was done using the *dredge* and *model.avg* functions in the *MuMIn* package in *R* (Bartoń, 2018).

## 3 RESULTS

All sampled oak trees were damaged by insect herbivores. Crown defoliation of focal trees (*T*_Def_) was on average 15.1% (*SE* ± 1.1) and ranged from 1% to 51%. At forest edge, *T*_Def_ was on average 22.3% (±3.0) and 11.7% (±1.5) in pure and mixed plots respectively, while in interior it was on average 16.9% (±2.7) and 14.7% (±1.8) in pure and mixed plots respectively. Leaf area removed by chewers ranged from 3% to 42% (mean = 13.4 ± 0.7%). On average, galls developed on 34.7% (±1.9) of sampled leaves, leaf miners on 22.1% (±0.9), sap feeders on 16.4% (±1.1), leaf tiers on 1.5% (±0.1) and leaf rollers on 0.4% (±0.1). Crown defoliation was positively and significantly correlated with leaf area removed by chewing herbivores (Pearson's correlations: *r* = 0.39, *p* < 0.001), and with the incidence of tiers and gallers (*r* = 0.22, *p* = 0.026 and *r* = 0.19, *p* = 0.044 respectively).

### 3.1 Effects of plot diversity and tree location on crown defoliation

**Figure 3:**
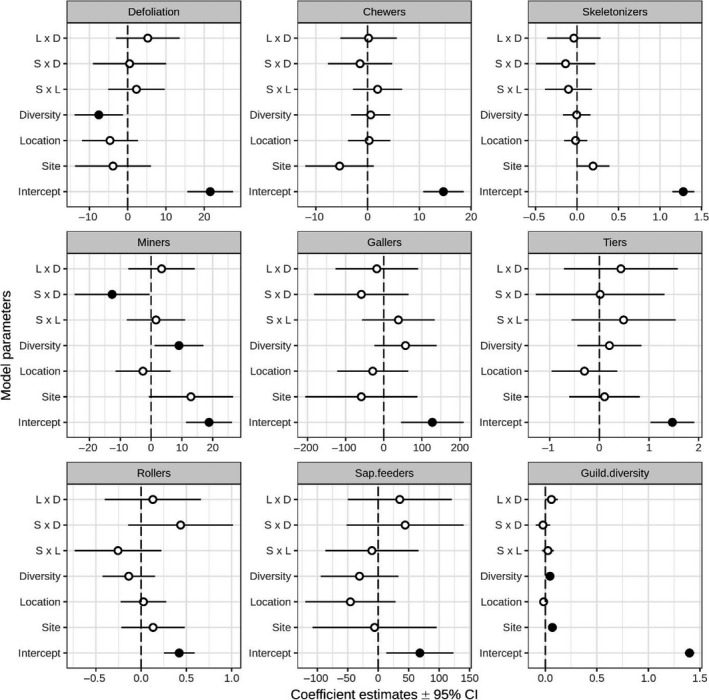
Model coefficient parameter estimates from the linear mixed models testing the effect of plot diversity (D: pure vs. mixed), plot location (L: edge vs. interior) and site (S: Aurignac vs. Lamothe) on total oak defoliation, guild diversity and guild‐specific damage or abundance. Parameters estimates are given for fixed effects of models within a Δ*_i_* = 2 units of the best model (i.e., the model with the lowest AICc). Patch identity is given as random factor. Black and white dots are significant and non‐significant predictors, as determined by 95% CI. For tree diversity, negative values indicate that the response variable was lower in mixed stands as compared to pure stands. For Location, negative values indicate that the response variable was lower at forest interior as compared to forest edges. For Site, negative values indicate that the response variable was lower at Lamothe as compared to Aurignac

**Figure 4:**
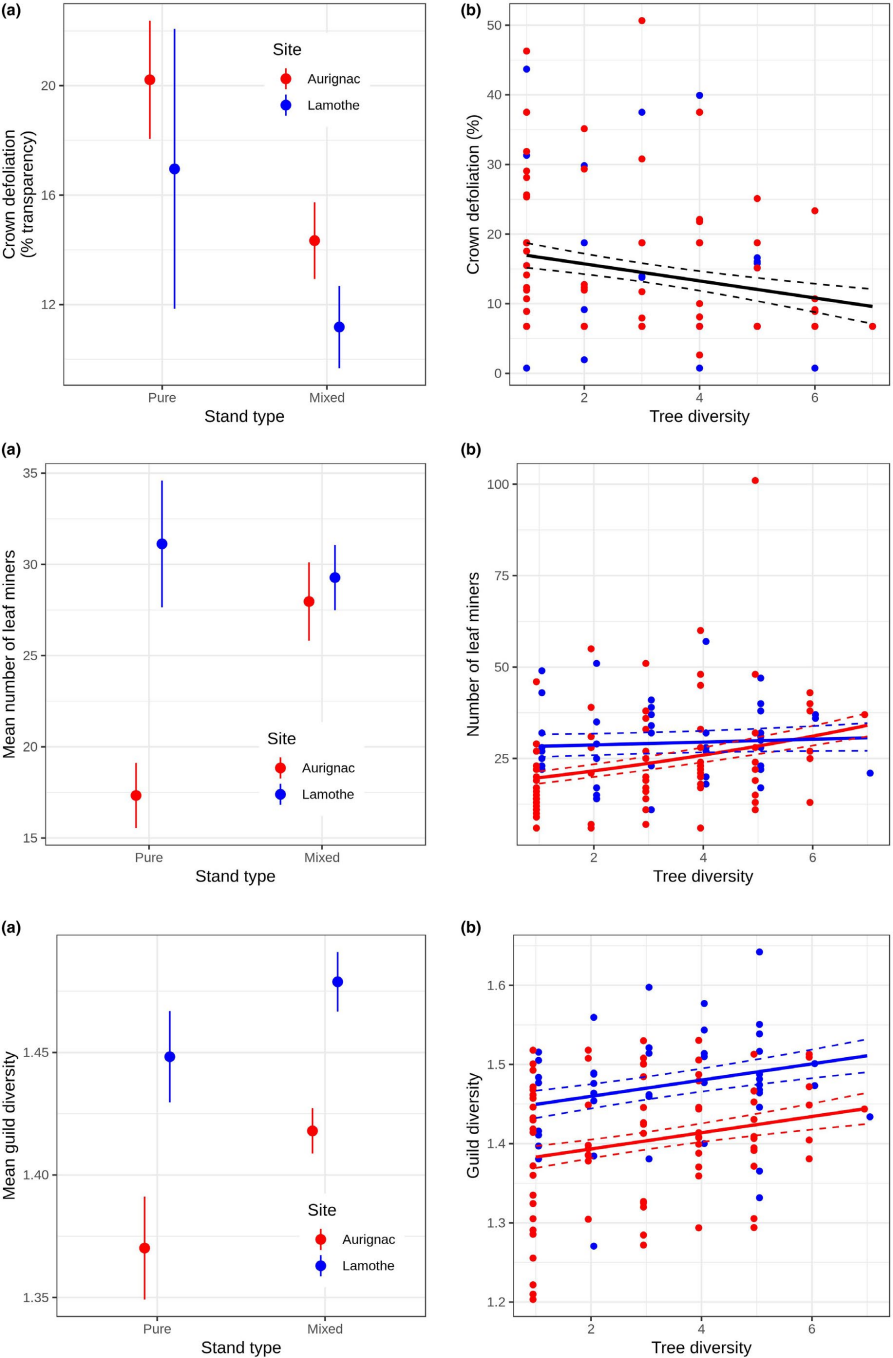
Effect of stand type (pure vs. mixed, A) and tree species richness (B) on (i) total tree crown defoliation, (ii) leaf miner abundance and (iii) herbivore guild diversity on focal oaks. In A, dots represent mean percentage of crown defoliation per focal oak tree (±*SE*). In B, dots represent individual focal oak tree. Solid lines and shaded areas represent predictions from the models and corresponding confidence intervals (95%)

When tree diversity was defined as pure versus mixed plots (i.e., stand type), the complete model was identified as the best model (i.e., with the lowest AICc), with no other competing model with ΔAICc < 2 (Table 2). However, the model coefficient parameters indicated that only stand type had a statistically clear effect on crown defoliation (Figure 3), whereby defoliation was on average lower in mixed plots than in to pure plots (Figure 4). Although retained in the best models, other predictors had no statistically clear effect on crown defoliation (Figure 3). This finding indicates that the overall effect of tree diversity on crown defoliation was consistent across sites and location within forests. The results were comparable when stand type was replaced by tree species richness to characterize tree diversity around focal oaks (Table 2, Figure 4) and consistently indicate that defoliation decreased with increasing tree species richness.

**Table 2:** Final selection of best linear mixed models testing the effect of plot tree diversity (pure vs. mixed or tree richness), location (edge vs. interior) and site on total oak defoliation, guild diversity and guild‐specific damage or abundance

Descriptor of tree diversity	Response	Model	AICc	Delta	Weight	*R* ^2^m	*R* ^2^c
Stand type (pure vs. mixed)	Defoliation	Location + Site + Stand type + Location × Site + Location × Stand type + Site × Stand type	913.17	0	0.52	0.08	0.23
	Chewers	Location + Site + Stand type + Location × Site + Location × Stand type + Site × Stand type	816.99	0	0.31	0.08	0.23
		Location + Site + Stand type + Location × Site + Site × Stand type	818.54	1.55	0.14	0.12	0.33
		Location + Site + Stand type + Location × Stand type + Site × Stand type	818.88	1.89	0.12	0.06	0.16
		Location + Site + Stand type + Location × Site + Location × Stand type	818.98	1.99	0.12	0.42	0.42
	Skeletonizers	Wood	131.17	0	0.58	0.08	0.23
		Site	132.53	1.36	0.29	0.12	0.33
	Miners	Location + Site + Stand type + Location × Site + Location × Stand type + Site × Stand type	970.59	0	0.64	0.08	0.23
	Gallers	Location + Site + Stand type + Location × Site + Location × Stand type + Site × Stand type	1,514.96	0	0.95	0.08	0.23
	Tiers	Wood	440.75	0	0.4	0.08	0.23
	Rollers	Wood	249.83	0	0.6	0.08	0.23
	Sap feeders	Location + Site + Stand type + Location × Site + Location × Stand type + Site × Stand type	1,451.45	0	0.93	0.08	0.23
	Guild diversity	Site	−260.1	0	0.44	0.08	0.23
		Site + Stand type	−258.9	1.19	0.24	0.12	0.33
		Wood	−258.13	1.97	0.16	0.06	0.16
Tree richness (1 to 7 sp.)	Defoliation	Location + Site +Tree richness + Location × Site + Location × Tree richness	922.01	0	0.26	0.08	0.2
		Location + Site + Tree richness + Location × Site + Location × Tree richness + Site × Tree richness	922.18	0.17	0.24	0.14	0.34
		Location + Site + Tree richness + Location × Site	923.62	1.61	0.12	0.09	0.21
		Location + Site + Tree richness + Location × Site + Site × Tree richness	923.74	1.73	0.11	0.36	0.36
	Chewers	Location + Site + Location × Site	820.83	0	0.3	0.08	0.2
		Location + Site + Tree richness + Location × Site + Site × Tree richness	822.56	1.74	0.12	0.14	0.34
	Skeletonizers	Wood	131.17	0	0.58	0.08	0.2
		Site	132.53	1.36	0.29	0.14	0.34
	Miners	Location + Site + Tree richness + Location × Site + Location × Tree richness + Site × Tree richness	979.12	0	0.46	0.08	0.2
	Gallers	Location + Site + Tree richness + Location × Site + Location × Tree richness + Site × Tree richness	1,521.49	0	0.89	0.08	0.2
	Tiers	Wood	440.75	0	0.47	0.08	0.2
	Rollers	Wood	249.83	0	0.7	0.08	0.2
	Sap feeders	Location + Site + Tree richness + Location × Site + Location × Tree richness + Site × Tree richness	1,458.38	0	0.84	0.08	0.2
	Guild diversity	Site	−260.1	0	0.66	0.08	0.2
		Wood	−258.13	1.97	0.25	0.14	0.34

Note

AICc, ΔAICc, weight, marginal (m) and conditional (c) *R* ^2^ are given for models within a Δ*_i_* = 2 units of the best model (i.e., the model with the lowest AICc). Patch identity (Wood) is given as random factor.

### 3.2 Variation of guild‐specific damage and guild diversity with plot diversity and location

Stand type or tree species richness, tree location or site had no statistically clear effects on guild‐specific damage or abundance, with the exception of leaf miners (Table 2, Figure 3). For leaf miners, the best model was the complete model, with no other competing model with ΔAICc < 2 (Table 2). Model coefficient parameter estimates indicated that stand type had a statistically clear effect on leaf‐mining herbivores that was contingent on site (i.e., Site × Diversity interaction). Specifically, leaf‐mining herbivores were more abundant in mixed stands than in pure stands; this effect was particularly strong in Aurignac site and was much weaker and opposite in Lamothe site (Figure 4a). However, replacing stand type by tree species richness to characterize tree diversity around focal oaks did not confirm the fact that tree diversity had a statistically clear effect on leaf miners (Figure 4b).

Guild diversity was significantly influenced by Stand type and Site, regardless of whether tree diversity was characterized by stand type or tree species richness (Table 2 and Figure 3). Specifically, guild diversity was greater in mixed stands than in pure stands and increased with tree species richness. These effects were consistent across sites, but the guild diversity was significantly lower in Aurignac than in Lamothe (Figure 4a,b).

## 4 DISCUSSION

We showed that in both sites, oaks were more defoliated in pure oak plots than in mixed plots at both edge and forest interior and that, on average, defoliation decreased with increasing tree diversity (1–7 species) demonstrating associational resistance patterns. However we also found that relationships between herbivory at leaf scale and tree diversity varied among insect feeding guilds and ranged from higher to equal in mixed plots as compared to pure plots. While damage made by some guilds differed between sites, they were independent of tree location at forest edges or interior. Herbivore guild diversity was also different between sites and increased with tree diversity whatever the oak location in both sites.

By considering both total crown defoliation and the leaf damage or insect incidence associated to seven herbivore feeding guilds, our study provides evidence for the debate on whether or not tree species diversity would lead to associational resistance in natural environments. While many reasons have been proposed to explain discrepancies in the literature, including insect herbivores' host specificity (Castagneyrol, Jactel, Vacher, et al., 2014; Jactel & Brockerhoff, 2007) or climatic conditions (Kambach, Kühn, Castagneyrol, & Bruelheide, 2016), the methodology of herbivory assessment may be another potential explanation. Indeed, in the present study focusing on oak species, we showed that tree diversity effects appear also to differ on guild‐specific leaf damages versus total crown defoliation. A reason may be that the total crown defoliation encompassed cumulative effects of many insect species since the beginning of the growing season, with potential opposite response of some guilds to tree diversity and tree location, whereas the guild‐specific damages were estimated at only one‐time point. Similarly, Sholes (2008) and Guyot et al. (2015) observed a significant decrease of insect damage in forests with higher tree diversity (*AR*) by evaluating final defoliation, on mature trees. By contrast, Schuldt et al. (2010) and Wein et al. (2016) observed higher herbivory damage in mixed forests by studying insect herbivory on individual leaves, in spring, on young saplings. Methodological issues like coarse assessment of overall crown defoliation versus more accurate estimates but on much fewer individual leaves may have also influenced the observed patterns.

Guild diversity increased with tree species diversity, most probably due to higher colonization success in more diverse tree communities (Liebhold et al., 2018). A higher number of insect species with different feeding habits (i.e., of different feeding guilds) are likely to locate, find and eventually colonize a suitable host tree within more diverse forests with trees of different size and qualities. And yet, because not all guilds cause similar amount of visible defoliation, higher herbivore diversity does not necessarily translate into higher crown damage.

The role of host specificity in dominant insect herbivores is known to be important in the response of herbivory to tree diversity, and it has been shown that *AR* is more likely to be observed against specialist than generalist insects (Castagneyrol, Jactel, Vacher, et al., 2014). However in our study, we found in general no significant effect of tree diversity on damage by each feeding guild, which might be due to the fact that we did not sample enough leaves to get a relevant estimate of their abundance. On the other hand, clear associational effects (being *AR* or *AS*) may be more likely to be observed when one focuses on abundance or damage made by a well identified herbivore species (e.g., Plath, Dorn, Riedel, Barriois, & Mody, 2012; Damien et al., 2016; Muiruri & Koricheva, 2016). On the contrary, when herbivory is assessed at the level of the herbivore community (e.g., total damage with no identification of responsible herbivore species), overall response to tree diversity might be blurred by opposite responses of different herbivore species. The only significant effect of tree diversity was observed on leaf miners in our study. Contrary to expectation, abundance of those herbivore specialists increased with forest diversity (associational susceptibility). One possible explanation for the difference with the theory is that we measured the number of leaf mines here, not the damage caused by leaf miners. Yet, the leaves may have accumulated mines made by several species, showing the same pattern of response to tree diversity as the diversity of herbivore guilds.

Our results provide no supporting evidence to the effect of tree location at forest edge or interior on herbivore‐plant interactions. This result confirms those recently found by van Schrojenstein Lantman et al. (2018) and Rossetti, Verena, Videla, Tscharntke, and Batary (2019), but contradicts Wirth et al. (2008) and Maguire et al. (2016) who showed that tree location can affect herbivory patterns. Numerous biotic and abiotic factors that can modify insect behavior or survival are acting at forest edge. Insects abundance and diversity are often higher at forest edge than in forest interior (Reitz & Trumble, 2002). Herbivore's natural enemies like predatory birds (Terraube et al., 2016) and insect parasitoids (Peralta, Frost, & Didham, 2018) also show strong response to forest edge effects. Trees at the ecotone between forest patches and open habitats are probably more sunlit but also more accessible by those insects, which migrate or move from one forest patch to another at each generation (Dulaurent et al., 2012; De Somviele, Lyytikainen‐Saarenmaa, & Niemela, 2007). A reason for the absence of edge effect in our study could be that the forest patches were too small, as edge effects can occur at kilometer‐scales for some taxa (Ewers & Didham, 2008). Previous results on highly variable responses of vegetation to edge effect in the same forest patches provide partial support to this hypothesis (Alignier & Deconchat, 2011). To better understand the processes that may cause different associational effects at the forest edge versus interior, it will be necessary to identify herbivorous species and characterize their biological traits (in particular diet specialization and dispersal abilities).

Finally, landscape‐mediated edge effects could also interact with forest interior conditions to influence ecological processes in forest patches (Garcia‐Romero, Vergara, Granados‐Pelaez, &, Santibanez‐Andrade, 2019). The site effects observed in our study suggest that the landscape context might specifically affect insect‐tree interactions as demonstrated by contrasting responses of leaf miners to forest diversity in the two study sites. Forest fragmentation can change the amount, quality and connectivity of habitat patches within a landscape (Hughes, Cobbold, Haynes, & Dwyer, 2015; Maguire et al., 2016). Our two studied sites belong to the same biogeographical area, but vary in their forest cover (18.5% vs. 9.2%). The amount of habitat and distances between habitat patches are known to influence metapopulation processes (Gilpin & Hanski, 1991) and hence the colonization probability of host trees by forest insect herbivores (Robert et al., 2018). Forest insect herbivory can be thus driven by a complex interplay between local tree diversity and stand isolation in the landscape (Castagneyrol, Giffard, Valdés‐Correcher, & Hampe, 2019).

## CONFLICT OF INTEREST

None declared.

## AUTHOR CONTRIBUTIONS

VG, HJ, MD and AV devised the conceptual idea of the study and designed the experimental sampling; VG, LB collected field data; WH designed the database; VG and BC conducted the statistical analyses; AV and VG led the writing of the manuscript; All coauthors made a significant contribution to the final manuscript.

## Data Availability

Herbivory data: GBIF https://doi.org/10.15468/nkxooz. https://www.gbif.org/dataset/25ff1a48-668d-470a-a149-5fa0e8535971
